# Essential genetic findings in neurodevelopmental disorders

**DOI:** 10.1186/s40246-019-0216-4

**Published:** 2019-07-09

**Authors:** Ana R. Cardoso, Mónica Lopes-Marques, Raquel M. Silva, Catarina Serrano, António Amorim, Maria J. Prata, Luísa Azevedo

**Affiliations:** 1 i3S - Instituto de Investigação e Inovação em Saúde, Population Genetics and Evolution Group, Universidade do Porto, Rua Alfredo Allen 208, 4200-135 Porto, Portugal; 2 IPATIMUP - Institute of Molecular Pathology and Immunology, University of Porto, Rua Júlio Amaral de Carvalho 45, 4200-135 Porto, Portugal; 3 Department of Biology, Faculty of Sciences, University of Porto, Rua do Campo Alegre, s/n, 4169-007 Porto, Portugal; 4 Department of Medical Sciences and iBiMED, University of Aveiro, Campus Universitário de Santiago, 3810-193 Aveiro, Portugal; 5 Present Address: Center for Interdisciplinary Research in Health (CIIS), Institute of Health Sciences (ICS), Universidade Católica Portuguesa, 3504-505 Viseu, Portugal

**Keywords:** Neurodevelopmental disorders, Brain-related genes, Deleterious mutations, de novo mutations, Polymorphisms, Risk alleles, Gene interaction

## Abstract

Neurodevelopmental disorders (NDDs) represent a growing medical challenge in modern societies. Ever-increasing sophisticated diagnostic tools have been continuously revealing a remarkably complex architecture that embraces genetic mutations of distinct types (chromosomal rearrangements, copy number variants, small indels, and nucleotide substitutions) with distinct frequencies in the population (common, rare, de novo). Such a network of interacting players creates difficulties in establishing rigorous genotype-phenotype correlations. Furthermore, individual lifestyles may also contribute to the severity of the symptoms fueling a large spectrum of gene-environment interactions that have a key role on the relationships between genotypes and phenotypes.

Herein, a review of the genetic discoveries related to NDDs is presented with the aim to provide useful general information for the medical community.

## Introduction

Neurodevelopment is the biological process resulting in the development and maturation of the nervous system. In humans, the process starts at the third week of embryonic growth with the formation of the neural tube [1–5]. From the ninth week onward, the brain orderly maturates and acquires its typical structure, under a tightly orchestrated chain of events that includes abundant cell proliferation, migration, and differentiation [1, 4, 5]. Any disruption to such orderly and complex chain of events may lead to dysfunctional brain development, and consequently to a neurodevelopmental phenotype. Under the designation neurodevelopmental disorders (NDDs) falls a group of complex and heterogeneous disorders showing symptoms associated to abnormal brain development that may give rise to impaired cognition, communication, adaptive behavior, and psychomotor skills [6–8]. NDDs include, for example, autism spectrum disorder, intellectual disability, attention deficit hyperactivity disorder, schizophrenia, and bipolar disorder [7, 9, 10]. The prevalence of these disorders constitutes a serious health problem in modern days. Previous reviews in distinct populations indicated a median global estimate of 62/10,000 for autism [11], 10.37/1000 for intellectual disability [12], and a median lifetime prevalence of 4/1000 for schizophrenia [13].

Multiple causes have been associated with NDDs, including genetic, environmental, infectious, and traumatic, among others, which in general do not operate alone but instead interacting between each other [6]. Importantly, the co-occurrence of distinct NDD entities has been often reported in the literature (e.g., [14]) suggesting the existence of shared underlying biological/cellular mechanisms [15, 16].

This review intends to focus on the molecular mechanisms associated with the most common neurodevelopmental illnesses, for which the precise etiology remains still largely unknown, but yet the genetic component has been increasingly deciphered with the massive sequencing of genomes of affected individuals.

### Gene/variant discovery by genome/exome screenings

Although the genetic etiology of NDDs is far from being completely known, significant advances have been made in the last years, achieved hand-in-hand with progresses in ascertaining specific biological pathways underlying the molecular mechanisms of these illnesses. The current mutational spectrum of NDDs includes many hundreds of genes related to neurodevelopmental pathways such as those associated with chromatin remodeling, synaptic function, and transcriptional regulation [17–19]. There is convincing evidence for the huge genetic heterogeneity not only within but also between and across different NDDs, once it is documented a considerable overlap of genes involved in more than one NDD, and the number of known causative genes continues to increase.

Whole exome sequencing (WES) has revealed to be among the most useful approaches in the identification of novel causal mutations [20–32] in particular WES-Trio (proband and parents) studies due to be based on the comparison of the genotypes of an affected child and their parents, allowing thus the identification both of de novo mutations as inherited risk variants with variable penetrance. The success of the WES approach was clearly demonstrated in a recent study based in consanguineous families with NDDs, in which 14 new candidate genes not previously associated with NDD disorders were identified (*GRM7*, *STX1A*, *CCAR2*, *EEF1D*, *GALNT2*, *SLC44A1*, *LRRIQ3*, *AMZ2*, *CLMN*, *SEC23IP*, *INIP*, *NARG2*, *FAM234B*, and *TRAP1*) all in patients who were homozygous for truncating mutations in each of the genes [31]. Importantly, the same study allowed the identification of a de novo dominant truncating mutation at the *PARD6A* (p.Arg312Term), a gene never yet associated with any human disease but whose mouse homolog had been demonstrated to control glial-guided neuronal migration [33]. Although future studies still need to address whether *PARD6A* plays a similar functional role present in humans, this illustrates the importance of WES in revealing new candidate genes that may have a critical role in the neurodevelopment.

Intronic mutations can also be identified through WES. In 2017, Prchalova et al. [34] reported on an adult female with severe intellectual disability, epilepsy, and autistic features among other symptoms in whom the WES analysis led to the detection of an intronic mutation in the *SYNGAP1* gene that was experimentally demonstrated to interfere with mRNA splicing. *SYNGAP1* encodes the Ras/Rap GTP-activating protein, which has a critical role in synaptic function [35, 36] and has been associated with NDDs [37].

Along with WES, whole genome sequencing (WGS) is further revealing the role of non-coding mutations in the development of NDD phenotypes, adding an extra dimension to the already complex etiology of these disorders [38–41]. Very recently, Short et al. [41] estimated that pathogenic de novo variants in fetal brain regulatory elements account for about 1–3% of exome-negative NDD probands. Therefore, WGS should be considered whenever exome analyses do not provide evidence regarding putative causative mutations in NDD phenotypes.

### Polymorphic variants and risk assessment

**Fig. 1:**
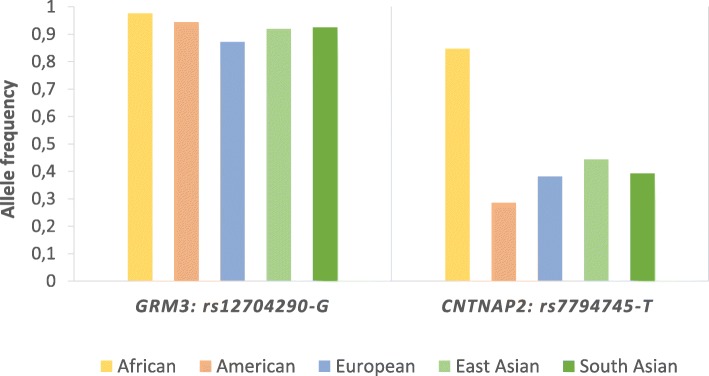
Risk allele frequency in five populations for two single nucleotide polymorphisms (SNPs) found to be associated to increased risk of neurodevelopmental disorders [47–51]. Data extracted from 1000 Genomes Project Phase 3 [52]

It is widely acknowledged that common genetic variations play an important role in the majority of complex disorders; actually, both rare and common alleles can contribute towards disease susceptibility [42]. Usually, variants with high frequency in the general population confer low relative risk [43, 44] while rare alleles highly penetrant may confer high risk [44]. Similarly to what is commonly found in other complex genetic disorders, the risk of developing NDDs seems to be highly influenced by the combined effect of common variants [45]. Up to now, thousands of common low-risk genetic variants that collectively can contribute to NDD susceptibility have been described [46]. Although the specific common risk alleles may differ between distinct NDDs, given their overall relevance here, we selected two single nucleotide polymorphisms (SNPs), highly polymorphic and showing replicated evidence of being associated with NDDs [47–51] to dissect their patterns of population distribution. In Fig. 1 is plotted the frequency of the assumed risk allele at each SNP across five major human populations.

The rs12704290 is an intronic variant located at *GRM3*, the gene that encodes the glutamate metabotropic receptor 3 involved in the glutamatergic neurotransmission. At this position, the assumed risk allele is rs12704290-G, which has been associated with a significant increased risk to schizophrenia [48, 50]. This allele is highly frequent across the five major human populations (Fig. 1), reaching the highest frequency in Africans (0.976) whereas the lowest is typically observed in Europeans (0.872).

The other common variant, rs7794745, is localized in the *CNTNAP2* gene, which encodes a neurexin family protein involved in cell-cell adhesion [53]. The allele rs7794745-T was previously associated with an increased risk of developing autism spectrum disorder [47, 49, 51] and is highly frequent in all human populations (Fig. 1). The presence of risk alleles showing high frequencies in different human populations led to the question on whether they were ancestral or derived alleles. To find the answer, we investigated which allele was present in the homologous positions in the available orthologous primate sequences using sequences available at the Ensembl project [54]. Interestingly, the two risk alleles (*GRM3* rs12704290-G and *CNTNAP2* rs7794745-T) were the ancestral configurations, a finding that likely explains the worldwide high frequencies both reach. According to di Rienzo and Hudson [55], cases in which the ancestral alleles contribute to increase risk to common diseases or disease-related phenotypes, whereas the derived alleles are protective, may have an evolutionary explanation whereby the ancestral alleles mainly reflect ancient adaptations of ancient human populations, but due to the environmental and lifestyle changes suffered in modern populations, such ancestral alleles become now variants that increase the risk of common diseases.

### Variants in chromatin-modifying/remodeling genes

The synaptic function may be influenced by many factors, among which are changes in chromatin dynamics caused by the disruption of a number of highly conserved genes [18]. Accordingly, chromatin-remodeling genes have been frequently reported in gene ontology analyses of data retrieved from WGS involving complex NDDs. For instance, *CHD2*, *CHD7*, and *CHD8*, three genes encoding chromodomain helicase DNA-binding (CHD) proteins that modulate chromatin structure, regulate gene expression, and play several other important roles, were previously linked to neurodevelopmental disorders such as intellectual disability [56]. Very recently, Kikawwa et al. [57] discussed the role played by the product of *PAX6* gene—Pax6, a chromatin modulator, in autism, reinforcing the importance of chromatin alterations in NDD genes.

### Clinical relevance of de novo mutations

De novo mutations are non-inherited sporadic mutations that arise either in the germline or in early embryonic development. As so, they do not conform to some rules of Mendelian inheritance, rendering more difficult to validate the prediction of their functional effect. When de novo mutations are associated with a clinical phenotype in a person without family history of a given condition, they can contribute to sporadic cases of the disease, including NDDs [58]. The de novo mutational rate of the human genome is approximately 1–3 × 10^− 8^ per base per generation [19, 59, 60] being well known that this rate is influenced by several factors, among which is the parental age [61]. The number of de novo mutations associated with NDDs has increased due to the strong investment in large-scale genetic screenings (exonic or genomic) of patients, which facilitate the identification of all types of molecular lesions as copy number variants (CNVs), indels, and mutations that cause gene disruption (missense, frameshift, and loss of splice site) [8, 62–68]. These spontaneous mutations were often found in candidate protein-coding genes with a high degree of haploinsufficiency or in regulatory elements involved in alternative splicing, in transcriptional regulation (enhancer and promoter), and in conserved non-coding sequences [41]. For example, in autistic patients, several de novo mutations were independently identified in the autism-associated genes *ADNP*, *ARID1B*, *CHD8*, and *SYNGAP1* [18, 69–71] revealing a likely deleterious effect. De novo mutations have also been identified in *GATAD2B* [72], *SCN2A* [73], and *FBXO11* [74] genes associated to intellectual disability, and in *PTPRG*, *TGM5*, *SLC39A13*, *BTK*, and *CDKN3* linked to schizophrenia [64].

Some mutations overlap distinct neurodevelopmental disorders [14, 75]. Accordingly, a WES-Trios study with schizophrenic patients conducted by McCarthy et al. [76] suggested a shared genetic etiology between schizophrenia, autism, and intellectual disability. Although the complete set of genes involved in NDD is far being from fully characterized, the recurrent identification of de novo mutations in a shared set of genes may allow further clarification and delineation of the molecular pathways that underlie NDDs. Also, experimental/functional validation of the identified de novo mutations is essential to separate disease-causing alleles from neutral variation.

### Genetic interaction

Genetic interaction (or epistasis) between genes or within the same gene is a major determinant of genotype-phenotype correlations [77–83]. The net result of distinct combinations of variants can result in distinct severities of the disease. Epistatic interactions between alleles are known for some Mendelian diseases revealing the interplay between mutations and polymorphisms which result in distinct functional outcomes [84, 85]. In what concerns the genetically heterogeneous neurodevelopmental disorders, the impact of the interaction between distinct alleles within the same *locus* or between interacting *loci* seems now to be giving its first steps. Evidence is emerging on intermolecular epistasis in autism spectrum disorders [86] regarding intramolecular and intermolecular epistasis between variants in the *SHANK2* family that were very recently documented [87]. This is in accordance with previous observations on the cumulative effect of disease-associated alleles in modulating neurodevelopmental phenotypes [88].

Variants in the sodium channel gene *SCN2A* have been often described in cohorts of patients with NDDs [89–91]. Among them is the common rs10174400-T allele, associated to impairment of cognitive ability in schizophrenic patients [92, 93] but with an unlikely effect in healthy individuals, which points towards a pathogenic effect that is conditionally dependent on the genetic background and, therefore, on the cumulative effect of distinct alleles as mentioned above. Extending these promising results to other neurodevelopmental disorders, it is expected that more cases of allelic interaction could highlight the etiology of these diseases, further explaining the genotype-phenotype correlation and the genetic overlap often observed [94].

## Conclusions

Neurodevelopmental disorders are a public health challenge due to complexity and heterogeneity of the etiology in conjugation with the high prevalence attained. Several biological pathways are disrupted in neurodevelopmental disorders, mainly at genes involved in synaptogenesis, chromatin remodeling, cell proliferation, and differentiation. Many of these genes, expressed during brain embryonic development, are intolerant to haploinsufficiency. It is important to continue the collection of information provided by WES and WGS data and focus deeply on epistatic interactions between identified mutations and polymorphic variants. In a more ambitious perspective, epigenetics may reveal itself as a promising therapeutic approach in the near future, exploiting the promise of numerous epigenome-wide association studies that are addressing neurodevelopmental disorders. Finally, it cannot be devaluated the major role that gene-environment interactions play in the outcomes of the diseases, implying that much attention should be given in the future to implement measures able to promote NDD prevention.

## Data Availability

Not applicable.

## Abbreviations

CHD: Chromodomain helicase deoxyribonucleic acid binding
CNV: Copy number variant
NDD: Neurodevelopmental disorder
SNP: Single nucleotide polymorphism
WES: Whole exome sequencing
WGS: Whole genome sequencing
